# Effects of Forming Lactoferrin–Milk Protein Complexes on Lactoferrin Functionality and Intestinal Development in Infancy

**DOI:** 10.3390/nu16234077

**Published:** 2024-11-27

**Authors:** Rulan Jiang, Xiaogu Du, Bo Lönnerdal

**Affiliations:** Department of Nutrition, University of California, Davis, CA 95616, USA

**Keywords:** lactoferrin, α-lactalbumin, protein complex, functionality of lactoferrin

## Abstract

Background/Objectives: Lactoferrin (Lf) is an iron-binding glycoprotein with multiple bioactivities, including promotion of cell proliferation and differentiation, immunomodulation, and antimicrobial activity. Lf, a basic glycoprotein, can bind to α-lactalbumin (α-Lac), an acidic whey protein. The current study aimed to evaluate whether Lf forms protein complexes with α-Lac and proteins/peptides from whey protein hydrolysate (WPH) and nonfat bovine milk powder (MP) and whether forming protein complexes influences resistance to gastrointestinal digestion and affects the bioactivities of Lf in human intestinal epithelial cells (HIECs and differentiated Caco-2 cells). Methods: Lf was blended with α-Lac, WPH, or MP. Assays were conducted to evaluate the bioactivities of proteins (Lf, α-Lac, WPH, or MP) and Lf–protein blends on HIECs and Caco-2 cells. Results: (1) Lf forms complexes with α-Lac and proteins/peptides from WPH and MP; (2) compared with Lf alone, complexed Lf shows greater resistance to in vitro digestion; (3) forming protein complexes does not affect Lf’s binding to the Lf receptor or its uptake by HIECs; and (4) forming protein complexes does not impact Lf’s bioactivities, including the promotion of cell proliferation and differentiation, reduction of cell permeability by upregulating tight-junction proteins, immune modulation through the regulation of IL-18, inhibition of enteropathogenic *Escherichia coli* growth, and modulation of immune responses to EPEC infection. Conclusions: Lf forms complexes with α-Lac and other milk proteins/peptides from WPH and MP in protein blends, and forming complexes does not affect the functionalities of Lf.

## 1. Introduction

Lactoferrin (Lf) is an iron-binding glycoprotein capable of binding two ferric ions [1]. Lf is found at high concentrations in human milk (1–7 g/L) [2], whereas its concentration in bovine milk is significantly lower (0.03–0.1 g/L) [3]. Lf exhibits a variety of bioactivities, such as upregulation of cell proliferation and differentiation, immunomodulation, and antimicrobial activity [4]. An intestinal Lf receptor (LfR) has been cloned and characterized [5]. Lf exerts many of its functions by binding to the Lf receptor (LfR) on the cell membrane, which is followed by the activation of cell signaling pathways and/or Lf functioning as a transcription factor after internalization and localization to the nucleus [6].

α-lactalbumin (α-Lac), a small Ca^2+^ binding protein (14.2 kDa), is the predominant whey protein in human milk, constituting approximately 25%–35% of total milk protein and 41% of the whey protein. Compared with its concentration in human milk, the α-Lac concentration in bovine milk is significantly lower, at around 2.5% of total milk protein. α-Lac contains abundant essential amino acids, including tryptophan and branched-chain amino acids [7]. In addition, it has been reported that α-Lac exerts a wide variety of biological activities, including pro-proliferation [8], as well as antioxidant [9], antitumor [10,11], antiviral [12], antimicrobial [13], anti-inflammatory [14,15], and neuroprotective properties [10].

Lf is a basic glycoprotein (pI~9.0) containing positive charges, and it is known to bind to acidic milk proteins, such as α-Lac (pI~4.5) [16], osteopontin (OPN, pI~3.6) [17], and β-lactoglobulin (pI~5.2) [18,19]. The bovine Lf–OPN complex has been shown to improve Lf resistance to in vitro simulated gastrointestinal digestion and to positively impact the proliferation of human intestinal cells compared with Lf and OPN alone [20]. Nevertheless, it is unclear whether the Lf–α-Lac complex or other Lf–protein/peptide complexes are functional and whether forming complexes influences functionality of Lf.

Human milk is the optimal food for infants. Infant formula is the appropriate substitute when human milk is not available. Nonfat bovine milk powder (MP) and bovine milk whey protein hydrolysate (WPH) are generally used as protein sources for infant formulas [21]. In the current study, we aimed to assess whether Lf forms protein complexes with α-Lac and proteins/peptides from MP and WPH, whether forming protein complexes affects resistance of Lf to gastrointestinal digestion, whether forming protein complexes influences binding of Lf to the Lf receptor on the cell membrane of intestinal epithelial cells, and whether the protein complexes exert similar bioactivities on intestinal epithelial cells as Lf.

We used two cell models, normal human intestinal crypt-like epithelial cells (HIECs) [22] and human enterocytes (differentiated Caco-2 cells) [23], to investigate the effects of various protein blends composed of Lf, α-Lac, MP, and/or WPH on intestinal development.

## 2. Materials and Methods

### 2.1. Preparation of Protein Blends

Bovine milk Lf (Bioferrin 1000, Glanbia Nutritionals, Twin Falls, ID, USA) was blended with bovine milk α-lactalbumin (α-Lac, Lacprodan Alpha-10, Arla Foods, Slagelse, Denmark), bovine milk whey protein hydrolysate (WPH, Hilmar 8350, Hilmar Ingredients, Hilmar, CA, USA), and/or bovine nonfat milk powder (MP, Organic Valley, LaFarge, WI, USA) to form three blends with different protein proportions (Table 1). MP was intentionally chosen for this investigation to ensure that the impact of MP proteins was studied without the impact of naturally occurring fats in MP. The endotoxin levels of proteins and protein blends were measured by using a Pierce™ Chromogenic Endotoxin Quant Kit (Thermo Fisher Scientific, Waltham, MA, USA) according to the manufacturer’s instructions. A pilot study was conducted to optimize concentrations for all experiments, so only one optimal concentration was used in each experiment.

### 2.2. Blue Native Polyacrylamide Gel Electrophoresis (PAGE) and Immunoblotting

Protein complexes in protein blends were evaluated using native PAGE. Native Bis-Tris gels (4−16%, Thermo Fisher Scientific) and the NativePAGE Novex Bis-Tris Gel System (Thermo Fisher Scientific) were used, followed by Coomassie brilliant blue R-250 (Sigma-Aldrich, St. Louis, MO, USA) staining according to the manufacturers’ instructions. Proteins were subsequently transferred from the native gel to polyvinylidene difluoride (PVDF) membranes using the Turbo Blotting System (Bio-Rad, Hercules, CA, USA). After membranes were blocked using a blocking solution (3% bovine serum albumin in TBST (Tris-buffered saline with 1% Tween-20)] at room temperature for 1 h, membranes were incubated sequentially with primary antibodies (anti-Lf, A10-126A, Bethyl Laboratories, Montgomery, TX, USA; anti-α-Lac, A10-125A, Bethyl Laboratories) and secondary antibodies (horseradish peroxidase-conjugated anti-goat IgG (Invitrogen, Carlsbad, CA, USA) or horseradish peroxidase-conjugated anti-rabbit (Cell Signaling Technology, Beverly, MA, USA)) in blocking solution at room temperature for 1 h. ProSignal Femto ECL Reagent (Genesee Scientific, San Diego, CA, USA) was used to visualize the proteins. The ChemiDocTM MP Imaging System (Bio-Rad) and the optimal exposure setting were used to generate all the images. All of the raw images were analyzed by ImageLab (Version 6.1) (Bio-Rad) and ImageJ (Version1.50d) (NIH) software.

### 2.3. In Vitro Digestion

To simulate gastrointestinal digestion, Lf, α-Lac, WPH, MP, and 3 Lf–protein blends were digested with gastric and intestinal digestion enzymes in vitro [24]. For mimicking infant gastric digestion, porcine pepsin (2% in 1 mmol/L HCl, 1:12.5 ratio of pepsin to protein; Sigma-Aldrich) was added to the samples (1 mg/mL in water) at pH 4.0, and samples were then incubated in an incubator (120–140 rpm) at 37 °C for 30 min. The pH was then adjusted to 7.0 with NaHCO_3_ (1 mol/L), after which pancreatin (0.4% in 0.1 mol/L NaHCO_3_, at a 1:62.5 ratio of pancreatin to protein; Sigma-Aldrich) was added. Samples underwent further incubation at 37 °C (120–140 rpm) for an additional 30 min. After incubation, pancreatin was inactivated by placing the samples in an 85 °C water bath for 3 min. To assess the impact of pH changes and 37 °C incubation on the proteins, samples without enzyme treatments were included for comparison. Afterwards, proteins with and without in vitro digestion were mixed with native loading buffer for blue native PAGE or Laemmli sample buffer (Bio-Rad) with β-mercaptoethanol (5%) followed by boiling for 5 min for SDS-PAGE. Samples were subjected to blue native PAGE or SDS-PAGE followed by Coomassie brilliant blue R-250 staining or Western blotting.

### 2.4. Cell Culture

Non-transformed human crypt-like intestinal epithelial cells (HIECs), a gift from Dr. Jean-François Beaulieu (Université de Sherbrooke, Sherbrooke, QC, Canada) [22], were cultured in Opti MEM (Life Technologies Inc., Gaithersburg, MD, USA) supplemented with fetal bovine serum (FBS, 5%, Gemini Bio-products, West Sacramento, CA, USA), NaHCO_3_ (2.4 g/L; Sigma-Aldrich), penicillin–streptomycin solution (0.5%; Gemini Bio-products), HEPES buffer (1%; Thermo Fisher Scientific), GlutaMax (1%, Thermo Fisher Scientific), and epidermal growth factor (5 ng/mL, Gemini Bio-products) and maintained in a humidified incubator at 37 °C under an atmosphere of CO_2_ (5%). The medium was changed every other day, and cells between passages 18–26 were used in the current study.

Human colon adenocarcinoma Caco-2 cells [American Type culture collection (ATCC), Rockville, MD, USA] were cultured in minimal essential medium (Gibco Invitrogen) containing FBS (10%, Gemini Bio-products), NaHCO_3_ (2.2 g/L, Sigma-Aldrich), and penicillin–streptomycin solution (1%, Gemini Bio-products) and maintained in a humidified incubator at 37 °C under an atmosphere of CO_2_ (5%). Passages 20–30 were used in the present study. After Caco-2 cells were delivered from ATCC, the passage number of the received cells was defined as 1.

### 2.5. Binding and Internalization of Lf in HIECs

Confocal microscopy was performed to assess whether Lf alone or in protein blends binds to the LfR on the membrane of intestinal epithelial cells and is then internalized by intestinal epithelial cells. HIECs (approximately 30–40% confluence) were seeded and cultured overnight on gelatin-coated glass coverslips (Neuvitro Corporation, Camas, WA, USA) in 24-well plates, with three wells per treatment. Colocalization was analyzed using ImageJ software [25]. After treatment with Lf or each protein blend in serum-free medium (SFM) at 37 °C for 1 h, cells were rinsed with PBS and fixed with 4% phosphate-buffered paraformaldehyde (0.4 mL/well) at room temperature for 10 min. Cells were then blocked with blocking buffer (5% heat-inactivated rabbit serum in PBS, 0.5 mL/well) for 20 min. To detect proteins (Lfs) and receptors (LfRs), cells were incubated with primary antibodies (anti-Lf from Bethyl Laboratories or anti-LfR [26]), followed by fluorescence-conjugated secondary antibodies (Alexa 488-conjugated or Fluor-633-conjugated antibodies; Invitrogen). After several PBS rinses, coverslips were mounted with ProLong Gold anti-fade reagent (Invitrogen), placed on glass slides, and sealed with nail polish. Immunofluorescence images were obtained using a confocal laser scanning microscope (FV1000, Olympus America Inc., Melville, NY, USA) with 60× magnification under an oil immersion lens, and images were analyzed using an integrated image analysis software system (Olympus America Inc.) and ImageJ (Version1.50d) [25].

### 2.6. Proliferation and Differentiation Assays

For all experiments, Lf, α-Lac, WPH, MP, and each Lf–protein blend were diluted proportionally, meaning that when samples were diluted 10× or 5×, the final concentrations were 0.1× and 0.2×, respectively. To evaluate effects on proliferation, HIECs were treated with Lf, α-Lac, WPH, MP, or each Lf–protein blend (1× concentration, n = 5) for 24 h, followed by a BrdU cell proliferation assay (Millipore, Bedford, MA, USA) according to the manufacturer’s instructions. To assess effects on cell differentiation, Caco-2 cells (90% confluence) were fasted for 2 h in serum-free medium (SFM) and then incubated with the proteins or protein blends (0.05× concentration, n = 5) in cell culture medium containing 1% FBS at 37 °C for 72 h. Cell lysates were prepared in a cell homogenization buffer (PBS containing 0.1% Triton X-100 and 1× complete protease inhibitor cocktail; Roche, Indianapolis, IN, USA). Alkaline phosphatase activity, an indicator of intestinal differentiation, was measured using p-nitrophenyl phosphate as a substrate (Thermo Fisher Scientific).

### 2.7. Transepithelial Electrical Resistance (TEER) Measurement

To assess whether Lf, α-Lac, WPH, MP, and Lf–protein blends influence permeability and the expression of tight-junction proteins in human intestinal epithelial cells, polarized Caco-2 cells were used as an enterocyte model [23]. After culturing Caco-2 cells on Transwells for 14 days, cells were treated with individual proteins or Lf–protein blends (0.5× in SFM; n = 5) for up to 24 h, and TEER was measured using the Millicell Electrical Resistance System (Millipore). Effects on the transcription of tight-junction proteins (claudin-1, occludin, and ZO-1) were evaluated by quantitative real-time polymerase chain reaction (qRT-PCR) as described below.

### 2.8. RNA Extraction and qRT-PCR

Total RNA was isolated from Caco-2 cells using Trizol reagent (Invitrogen), treated with DNase I (New England Biolabs, Ipswich, MA, USA), purified using an RNA Clean & Concentrator-5 kit (Zymo Research, Irvine, CA, USA), and reverse-transcribed to cDNA using a High-Capacity cDNA Reverse Transcription Kit (Applied Biosystems, Foster City, CA, USA), following the manufacturers’ instructions. Gene-specific primers were designed using the NCBI primer design tool (Table 2) and ordered from Operon Technologies (Alameda, CA, USA). qRT-PCR was performed on 2 µL of the cDNA reaction mixture with SYBR green (Bio-Rad) using an iCycler Real-Time PCR System (Bio-Rad). The cycling parameters were 95 °C for 15 min, followed by 40 cycles of 95 °C for 15 s, 60 °C for 30 s, and 72 °C for 30 s. The linearity of the dissociation curve was analyzed using iCycler software (Version 3.1), and the mean cycle threshold (Ct) of the linear portion of the curve was designated as Ct. Each sample was analyzed in triplicate and normalized to GAPDH using the equation: fold change (treatment/control) = 2 ^(Ct control target gene − Ct control GAPDH) − (Ct treatment target gene − Ct treatment GAPDH)^. The results are expressed as the mean fold change ± standard deviation, relative to the control (set to 1).

### 2.9. IL-18 Secretion

Caco-2 cells (day 14) were fasted for 2 h in serum-free medium (SFM) and then treated with Lf, α-Lac, WPH, MP, or each Lf–protein blend in cell culture medium containing 1% FBS at 37 °C for 72 h. The cell culture media were collected and concentrated using YM-3 filters (3K MWCO; Millipore). Secreted interleukin-18 (IL-18) levels in the cell culture media were measured with an ELISA kit (R&D Systems, Minneapolis, MN, USA) according to the manufacturer’s instructions.

### 2.10. Effects on Transcription of the TGF-β1 Gene

Caco-2 cells (day 8) on 6-well plates were treated with Lf, α-Lac, WPH, MP, or Lf–protein blends (0.1×) at 37 °C for 72 h. RNA extraction and qRT-PCR processing were performed as described above.

### 2.11. Effects on Growth of Enteropathogenic Escherichia coli (EPEC)

The effects of the proteins and protein blends on the growth of enteropathogenic *E. coli* (EPEC) (BAA-2440, ATCC) were examined by counting colony-forming units (CFUs) on agar plates. Samples of EPEC (5 × 10^5^ CFUs/mL) were cultured with Lf, α-Lac, WPH, MP, or Lf–protein blends (0.5× of each in Difco Nutrient Broth medium) and incubated at 37 °C with shaking at 225 rpm for 16 h. Aliquots of the medium (10 μL) were plated onto Luria−Bertani (LB) agar, and CFUs were counted after overnight incubation at 37 °C.

### 2.12. Effects of Proteins and Lf–Protein Blends on the Inflammatory Response Induced by EPEC Infection: IL-1β, IL-6, and TNF-α

Caco-2 cells (day 16) were fasted in SFM without antibiotics at 37 °C for 1 h and then incubated with EPEC (250 μL, optical density approximately 0.4 at 600 nm) and with Lf, WPH, MP, or Lf–protein blends (1× in SFM without antibiotics) at 37 °C for 4 h. After infection, cells were washed twice with PBS and then cultured with complete medium containing gentamicin (1 µg/mL) at 37 °C for 4 h. Caco-2 cells were subsequently collected for the RNA extraction and qRT-PCR analysis of pro-inflammatory cytokines (IL-1β, IL-6, and TNF-α) transcripts as described above. Primer sequences are provided in Table 2.

### 2.13. Statistical Analysis

Data are presented as the mean ± standard deviation from 2–3 independent experiments. Comparisons between control and treatment groups were conducted using Student’s *t*-test or one-way ANOVA tests (GraphPad Prism (Version 10), GraphPad Software Inc., San Diego, CA, USA). The means from different treatment groups were compared using Tukey’s honestly significant difference test. *p* < 0.05 was considered statistically significant.

## 3. Results

### 3.1. Endotoxin Levels of Proteins and Blends

**Figure 1:**
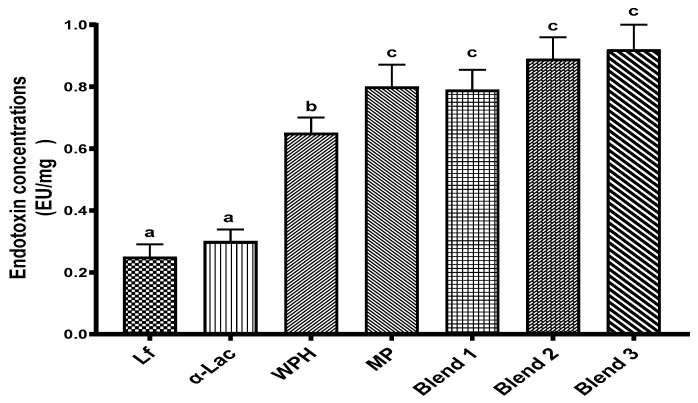
Endotoxin levels of proteins and protein blends. Results are shown as the mean ± SD. Bars without a common letter are significantly different (n = 3, *p* < 0.05). Abbreviations: Lf: lactoferrin; α-Lac: α-lactalbumin; WPH: whey protein hydrolysate; MP: bovine milk powder.

When Lf is added to bovine milk-based infant formulas, it interacts with proteins and peptides from the milk. To simulate the addition of Lf to infant formulas, the bioactivities of Lf alone, as well as Lf blended with α-Lac, whey protein hydrolysate, and/or nonfat milk powder, were evaluated (Table 1). The proportions of Lf and whey proteins are based on the composition of human milk [27] and pilot studies. As shown in Figure 1, all proteins and protein blends contain endotoxin, and all values are below 1 EU/mg.

### 3.2. In Vitro Digestion

**Figure 2:**
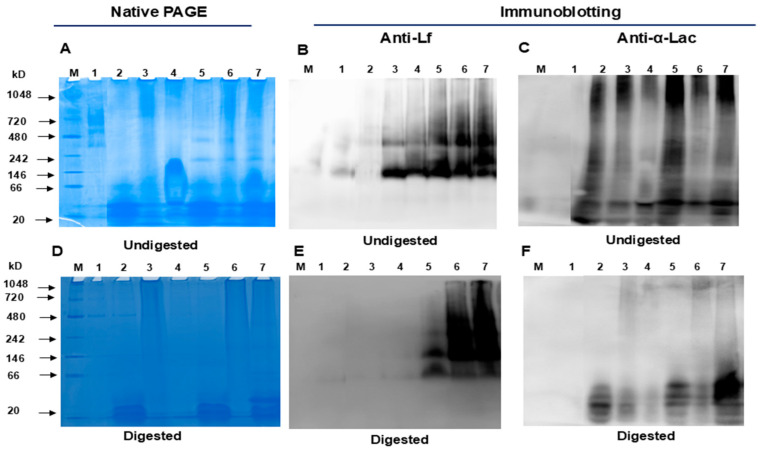
Native PAGE and Western blotting analysis of Lf, α-Lac, WPH, MP, and protein blends. Proteins and protein blends (1 mg/mL for each) were digested with pepsin and pancreatin for 30 min, respectively. Undigested and digested proteins or protein blends (10 µL for each) were subjected to native PAGE and stained with Coomassie brilliant blue R-250 or probed with antibodies (anti-Lf or anti-α-Lac). (**A**–**C**) non-digested Lf and blends; (**D**–**F**) digested Lf and blends. (**A**,**D**): Native PAGE; (**B**,**E**): Western blotting (anti-Lf); (**C**,**F**): Western blotting (anti- α-Lac). M: protein molecular marker; 1: Lf; 2: α-Lac; 3: WPH; 4: MP; 5: blend 1; 6: blend 2; 7: blend 3. Abbreviations: Lf: lactoferrin; α-Lac: α-lactalbumin; WPH: whey protein hydrolysate; MP: bovine milk powder.

To evaluate whether Lf forms complexes with α-Lac and other proteins/peptides in WPH and MP, blue native PAGE (Figure 2A) and native Western blotting (Figure 2B,C) were conducted. As shown in Figure 2A–C, the protein bands of Lf alone and α-Lac alone differ from bands of Lf and α-Lac in protein blends, indicating that Lf and α-Lac form a complex with each other and with milk proteins/peptides from WPH and MP. To evaluate whether Lf and Lf–protein complexes are resistant to in vitro simulated gastrointestinal digestion, these proteins were digested with pepsin at pH 4.0 and pancreatin at pH 7.0 at 37 °C for 30 min, respectively. The digested protein samples were then analyzed by blue native PAGE and native Western blotting, respectively. As shown in Figure 2D–F, both Lf and α-Lac are more resistant to in vitro digestion when complexed with each other or other proteins. Lf formed similar bands in undigested samples and digested samples.

**Figure 3:**
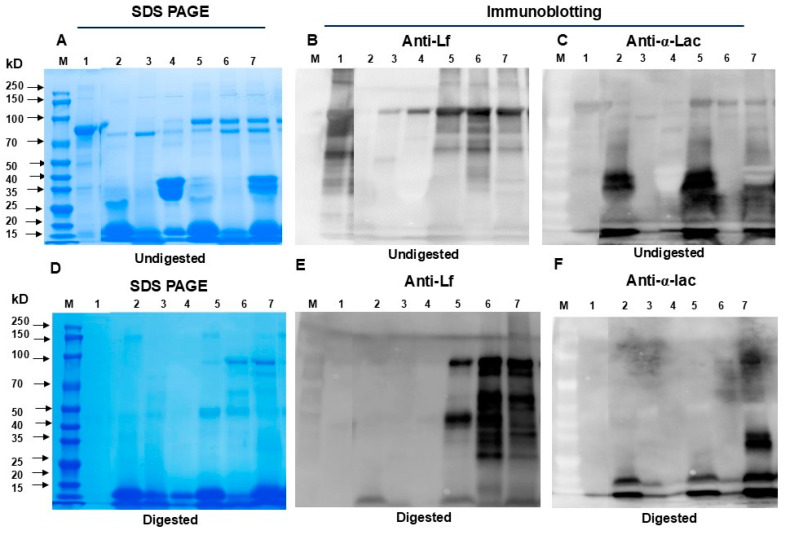
SDS PAGE and Western blotting analysis of Lf, α-Lac, WPH, MP, and protein blends. Proteins and protein blends (1 mg/mL for each) were digested with pepsin and pancreatin for 30 min, respectively. Undigested and digested proteins and protein blends (10 µL for each) were subjected to SDS PAGE and stained with Coomassie brilliant blue R-250 or probed with antibodies (anti-Lf or anti-α-Lac). (**A**–**C**) Non-digested proteins and protein blends; (**D**–**F**) digested Lf and blends. (**A**,**D**): SDS PAGE; (**B**,**E**): Western blotting (anti-Lf); (**C**,**F**): Western blotting (anti- α-Lac). M: protein molecular marker; 1: Lf; 2: α-Lac; 3: WPH; 4: MP; 5: blend 1; 6: blend 2; 7: blend 3. Abbreviations: Lf: lactoferrin; α-Lac: α-lactalbumin; WPH: whey protein hydrolysate; MP: bovine milk powder.

SDS PAGE and Western blotting were also performed before and after in vitro simulated gastrointestinal digestion (Figure 3A–F) to show the size (molecular weight) of Lf alone, α-Lac alone, and each in complexes with other proteins/peptides. The SDS PAGE analysis of the undigested proteins (Figure 3A–C) show similar molecular weights of Lf alone or in blends and α-Lac alone or in blends. Both Lf alone and α-Lac alone were partly resistant to in vitro gastrointestinal digestion, but they were relatively protected from digestion when in protein blends. Forming complexes with α-Lac and other proteins from WPH and MP protects Lf and α-Lac from digestion by gastric and intestinal enzymes (Figure 3D–F). The SDS PAGE and Western blotting images were modified because we decided to exclude certain treatments that were initially planned for inclusion in the manuscript. The raw images have been provided in the Supplementary Materials.

### 3.3. Binding of Lf to the LfR and Internalization by Intestinal Epithelial Cells

**Figure 4:**
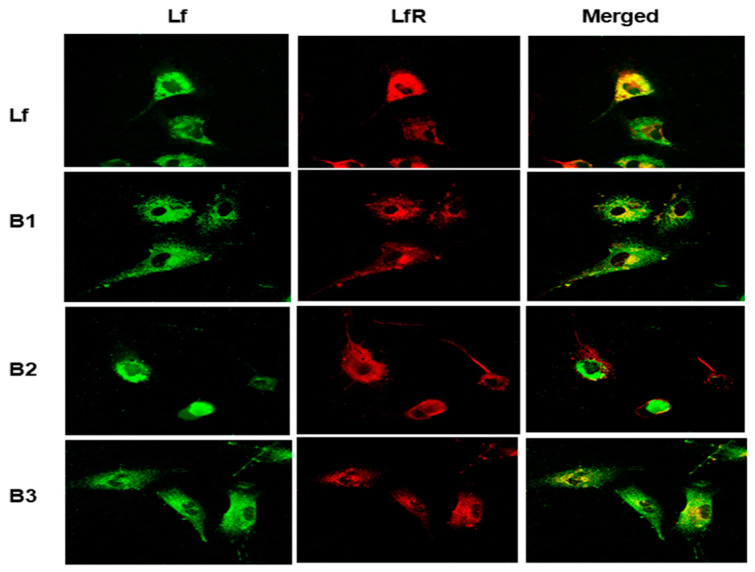
Binding and internalization of Lf in human intestinal epithelial cells (HIECs). HIECs grown on glass cover slips were treated with Lf and protein blends at 37 °C for 1 h, fixed, and then probed with rimary antibodies (anti-Lf or anti-LfR antibodies) and then fluorescent secondary antibodies. Lf (green) and LfR (red).

We have previously demonstrated that LfR mediates Lf internalization by intestinal epithelial cells via clathrin-dependent endocytosis [26]. We therefore used confocal microscopy to evaluate whether Lf alone and in complex forms is able to bind to the LfR on the cell membrane and is then internalized by intestinal epithelial cells. After HIECs were treated with Lf or Lf–protein blends at 37 °C for 1 h, HIECs were fixed for confocal microscopy analysis. Representative images are shown in Figure 4; both Lf alone and Lf in complex forms (in all the protein blends) were able to bind to the LfR and were subsequently internalized by HIECs. Colocalization was analyzed by using ImageJ software [25], and no significant differences were found among these treatment groups.

### 3.4. Effects of Lf, α-Lac, WPH, MP, and Lf–Protein Blends on Proliferation and Differentiation of Intestinal Epithelial Cells

**Figure 5:**
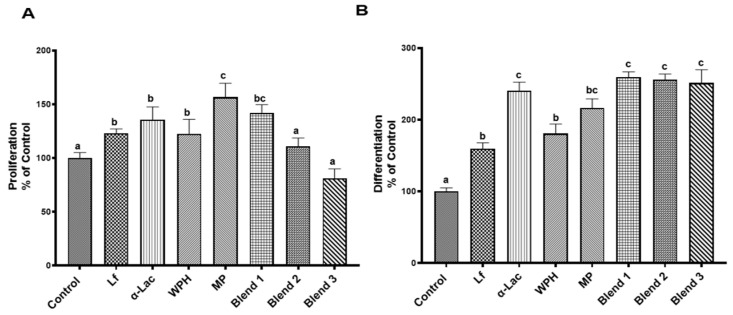
Effects of Lf, α-Lac, WPH, MP, and Lf–protein blends on proliferation of HIECs (**A**) and differentiation of Caco-2 cells (**B**). (**A**) HIECs were grown on 96-well plates (approximately 40% confluence) and were incubated with Lf, α-Lac, WPH, MP, or Lf–protein blends (1× of each) at 37 °C for 24 h. Cell proliferation was then evaluated by a BrdU ELISA kit. (**B**) Caco-2 cells grown on 24-well plates (90% confluence) were treated with Lf, α-Lac, WPH, MP, or Lf–protein blends (0.05× of each) at 37 °C for 72 h. Alkaline phosphatase activity, a cell differentiation marker, was measured by using pNPP as a substrate. Results are shown as the mean ± SD. Bars without a common letter are significantly different (n = 5, *p* < 0.05).

It is known that Lf promotes intestinal proliferation and differentiation [28]. HIECs were treated with Lf, α-Lac, WPH, MP, or Lf–protein blends (1× for each) at 37 °C for 24 h, and the BrdU assay was then carried out. As shown in Figure 5A, Lf, α-Lac, WPH, MP, and blend 1 markedly increased proliferation of HIECs. After 72 h treatment of Caco-2 cells with proteins and protein blends, the effects on the differentiation of Caco-2 cells were evaluated by measuring the activity of alkaline phosphatase, an intestinal differentiation marker [29]. As shown in Figure 5B, all proteins and blends promoted the differentiation of Caco-2 cells. All of the protein blends exhibited greater effects than Lf did alone.

### 3.5. Effects of Proteins and Protein Blends on Intestinal Barrier Functions

**Figure 6:**
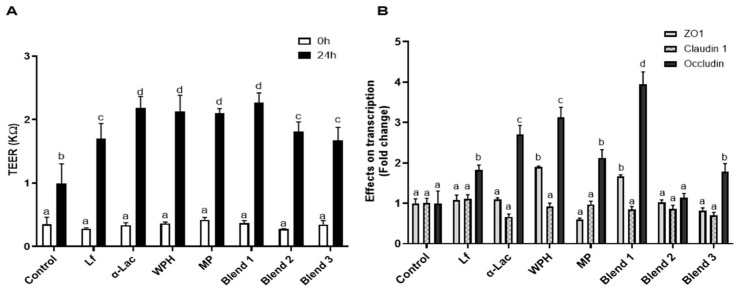
Effects of Lf, α-Lac, WPH, MP, and protein blends on TEER and transcription of tight junction proteins. (**A**) After D14 Caco-2 cells on Transwells were treated with proteins or Lf–protein blends (0.5× for each) at 37 °C for 48 h, TEER was measured. Results are shown as the mean ± SD. (**B**) Cells were collected for qRT-PCR. Results are shown as the mean fold change ± SD. Bars without a common letter are significantly different (n = 5, *p* < 0.05).

After Caco-2 cells were cultured on Transwells for 14 days, the cells were treated with Lf, α-Lac, WPH, MP, or Lf–protein blends (0.1× for each) at 37 °C for 48 h. Trans-epithelial electrical resistance (TEER) was measured at 24 h to determine the effects on intestinal barrier functions. All of the proteins and protein blends significantly increased TEER. α-Lac and Lf-α-Lac showed a greater increase in TEER than Lf did alone. Next, whether the proteins and blends enhanced TEER by influencing expression of tight-junction proteins was examined. As shown in Figure 6B, all proteins and blends (except blend 1) increased the transcription of occludin, some more than others. WPH and blend 1 also increased transcription of the ZO1 gene.

### 3.6. Effects of Proteins and Protein Blends on Transcription of the TGF-β1 Gene

**Figure 7:**
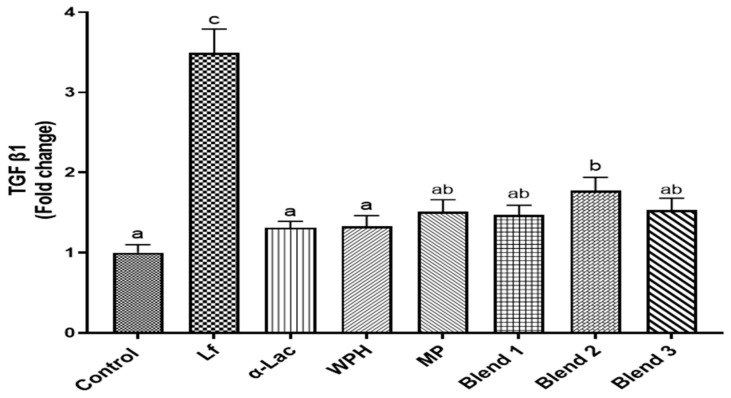
Effects of Lf, α-Lac, WPH, MP and Lf–protein blends on transcription of the TGF-β1 gene. After differentiated Caco-2 cells (D8) were treated with proteins or protein blends at 37 °C for 72 h, total RNA was extracted for qRT-PCR. Results are shown as the mean fold change ± SD. Bars labeled with different letters are significantly different (n = 5, *p* < 0.05).

Differentiated Caco-2 cells (D8) were cultured at 37 °C for 8 days and then treated with Lf, α-Lac, WPH, MP, or Lf–protein blends (0.5× for each) for 24 h. RNA was then extracted for qRT-PCR. Only Lf and blend 2 increased transcription of the TGF-β1 gene (Figure 7). The effect of Lf was the greatest. Although MP and three protein blends also increased transcription of the TGF-β1 gene, the results were not significantly different from the control.

### 3.7. Effects of Proteins and Protein Blends on IL-18 Secretion by Caco-2 Cells

**Figure 8:**
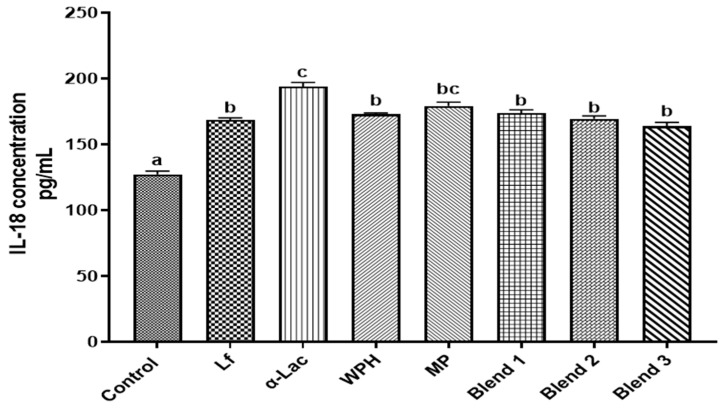
Effects of proteins and protein blends on IL-18 secretion by Caco-2 cells. After Caco-2 cells (D14) were treated with proteins or protein blends at 37 °C for 72 h, the cell culture medium was concentrated by YM-3 and then analyzed with an IL-18 ELISA kit. Results are shown as the mean ± SD. Bars labeled with different letters are significantly different (n = 5, *p* < 0.05).

IL-18 has multiple functions, including the enhancement of barrier homeostasis, and its effects vary depending on the context and location [30]. Lf has been shown to increase the expression of IL-18 in intestinal epithelial cells [31], making IL-18 a useful marker for assessing Lf’s impact on the immune response. Differentiated Caco-2 cells (D14) were treated with Lf, α-Lac, WPH, MP, or protein blends (0.1× for each) for 48 h, and cell culture media were collected for IL-18 quantification using ELISA. As shown in Figure 8, all proteins and protein blends markedly increased secreted IL-18 in cell culture media.

### 3.8. Effects of Proteins and Protein Blends on Growth of EPEC

**Figure 9:**
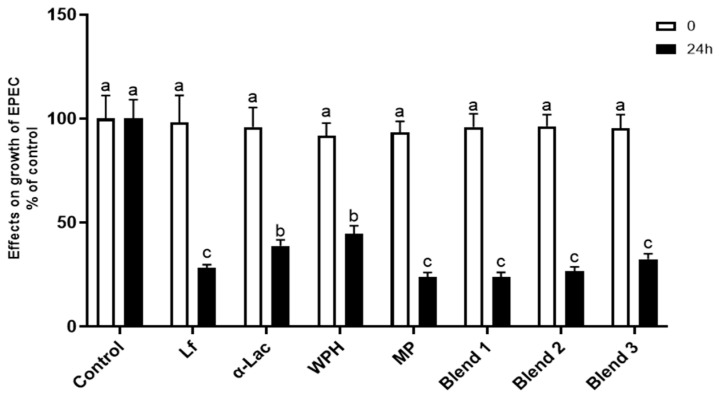
Effects of proteins and protein blends on the growth of EPEC. EPEC (5 × 10^5^ CFUs/mL) was cultured with proteins or protein blends (0.2× of each in Difco Nutrient Broth medium) with shaking (250 rpm) at 37 °C for 16 h. Aliquots of the medium (10 μL) were plated onto LB agar, and CFUs were counted after overnight incubation at 37 °C. Results are shown as the mean ± SD. Bars labeled with different letters are significantly different (n = 5, *p* < 0.05).

EPEC was cultured with Lf, α-Lac, WPH, MP, or Lf–protein blends (0.2× for each) at 37 °C for 16 h; the culture medium was then grown on agar plates; and EPEC colonies were then counted at 24 h. As shown in Figure 9, all proteins and protein blends significantly inhibited the growth of EPEC. α-Lac and WPH showed the least inhibition.

### 3.9. Effects of Proteins and Lf–Protein Blends on Immune Responses to EPEC Infection

**Figure 10:**
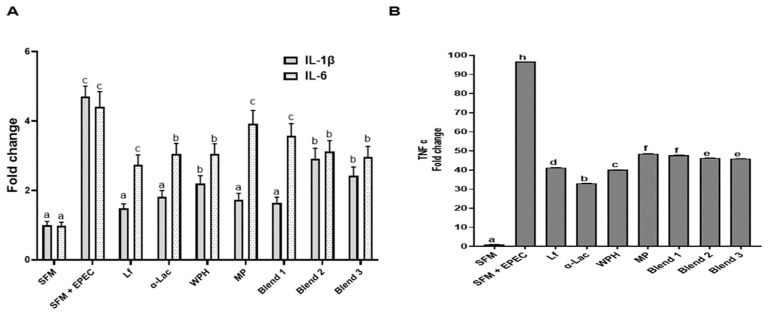
Effects of proteins and Lf–protein blends on the inflammatory response induced by EPEC infection. After Caco-2 cells (day 16) were incubated with EPEC (250 μL, optical density approximately 0.4 at 600 nm) and with proteins or protein blends at 37 °C for 4 h, cell culture media were removed, cells were rinsed with PBS, and cells were cultured in complete medium containing gentamicin (1 µg/mL) at 37 °C for 4 h. RNA from Caco-2 cells was then extracted for the qRT-PCR analysis of pro-inflammatory cytokine (IL-1β, IL-6 (**A**), and TNF-α (**B**)) transcripts. Bars labeled with different letters are significantly different (n = 5, *p* < 0.05).

After differentiated Caco-2 (D16, cultured in 6 well plates) were infected by EPEC and incubated with Lf, α-Lac, WPH, MP, or protein blends, the effects on immune responses of the cells to EPEC infection were evaluated. As shown in Figure 10, EPEC infection significantly increased the transcription of IL-1β, IL-6, and TNF-α genes. All proteins and protein blends inhibited the transcription of IL-1β and TNF-α to a different extent. All proteins and protein blends, except MP and blend 2, decreased transcription of IL-6.

## 4. Discussion

Lf forms complexes with α-Lac as well as with proteins/peptides from WPH and MP, and forming protein complexes protect Lf against in vitro simulated gastrointestinal digestion. It is likely that a portion of Lf binds to α-Lac as well as with proteins/peptides from WPH and MP. Proteins that are not bound to Lf act independently of the complexes, and the activity of the blends reflects the combined action of all whey or milk proteins. It is possible that forming protein complexes influences the interaction between Lf and receptors, such as the heparin sulfate receptor. However, Lf complexes may exhibit greater bioactivity than Lf alone through mechanisms that do not rely on the receptor-binding site. Lf alone was minimally resistant to in vitro gastrointestinal digestion, but a higher percentage of Lf in protein blends survived digestion probably by forming protein complexes with α-Lac and proteins and peptides from WPH and MP (Figure 2 and Figure 3), which suggests that Lf in protein blends may exert more potent beneficial effects on the intestine in early life. This would be similar to human milk, in which Lf can bind to various other milk proteins and limit digestibility and preserve Lf functionality. In agreement with the findings from this current study, Lf complexed with OPN or Lf blended with OPN, α-Lac, and WPH exhibited greater resistance to gastrointestinal digestion than Lf alone [32].

Forming Lf–milk protein complexes did not influence the binding of Lf to the LfR or internalization by intestinal epithelial cells but affected various bioactivities of Lf. As shown in Figure 4, Lf alone and in three protein blends were able to bind to the LfR on the cell membrane of HIECs. Lf exerts multiple functions by binding to the LfR, followed by initiation of cell signaling pathways or translocation to the nucleus to regulate gene transcription [6]. Lf promotes the proliferation and differentiation of intestinal epithelial cells, lowers the cell permeability of intestinal epithelial cells by upregulating expression of tight-junction proteins, regulates immune functions, as well as inhibits the growth of EPEC and regulates immune responses to EPEC infection. Lf and protein blends varied in their effects on proliferation, differentiation, and transcription of the TGF-β1 gene. To compare bioactivities of Lf and protein blends, the same dilution of Lf and protein blends were used for all of the assays. The differing concentrations of total protein(s) in these preparations may have affected results in specific assays. WPH and MP exhibit many bioactivities similar to Lf, but they did not promote transcription of the TGF-β1 gene. The protein concentration of WPH and MP used in the current study was much higher than that of Lf, which is usually the case in infant formula. WPH and MP consist of diverse protein components, such as β-lactoglobulin, immunoglobulins, and lactoperoxidase [33,34,35]. Lf may have a more potent effect on gene transcription of TGF-β1 than other bioactive components in whey proteins.

α-Lac is relatively resistant to in vitro simulated gastrointestinal digestion and exhibits beneficial effects on intestinal epithelial cells. Both α-Lac alone and α-Lac in protein blends are partly resistant to in vitro simulated gastrointestinal digestion. This is in agreement with previous studies showing that α-Lac in human milk and infant formula is relatively resistant to in vitro digestion [36,37]. α-Lac and Lf as well as peptides/proteins from WPH and MP form protein complexes, as shown in Figure 1 and Figure 2. Studies on the bioactivities of α-Lac have been comparatively limited and have largely focused on its peptides, but we show that α-Lac exhibits multiple effects on intestinal epithelial cells, including the promotion of cell proliferation and differentiation, decrease in cell permeability, increase in expression of a tight-junction protein, inhibition of the growth of EPEC, and regulation of immunity of intestinal epithelial cells. Our results are consistent with some in a previous study that showed that α-Lac increased TEER in differentiated Caco-2 cells [38] and effects on IL-1 β and TNF-α secretion [15].

There are limitations of the present study. As mRNA levels are not always correlated with protein expression due to several reasons, such as the post-transcriptional modification, protein levels instead of mRNA levels would be a better indicator to show effects of proteins and protein blends on effects on TGF-β1, IL-1β, IL-6, and TNF-α.

## 5. Conclusions

In summary, Lf forms complexes with α-Lac and other milk proteins/peptides from WPH and MP in protein blends. Compared with Lf alone, Lf in complex forms is more resistant to in vitro simulated gastrointestinal digestion, suggesting that Lf in complex forms may play important roles in intestinal development. Forming Lf–protein complexes does not influence binding of Lf to LfR and uptake of Lf by intestinal epithelial cells. Lf, α-Lac, and protein blends exhibit beneficial effects on intestinal epithelial cells including the promotion of proliferation and differentiation, lowering of cell permeability by increasing expression of tight-junction proteins, regulation of immune functions by regulating IL-18, and inhibition of the growth of EPEC as well as modification of immune responses to EPEC infection. Our finding that α-Lac stimulated cell proliferation and differentiation, TEER, and occludin as well as IL-18 expression was somewhat unexpected. It is known, however, that breast-fed infants have a well developed and differentiated small intestine, and it is thus possible that the high concentration of α-Lac in breast milk is in part responsible for this. These activities of α-Lac merit further studies.

## Figures and Tables

**Table 1:** Protein proportions.

	Lf	α-Lac	WPH	MP
Blend 1	1	19.2		
Blend 2	1		30.16	
Blend 3	1	19.2	30.16	29.04

**Table 2:** Primers for qRT-PCR reactions.

	Primer Sequence	5′-3′	
Gene	Forward	Reverse	Accession Number
Claudin-1	CTGGGAGGTGCCCTACTTTG	GGCCTTGGTGTTGGGTAAGA	NM_021101.5
Occludin	AAGGTTCCATCCGAAGCAGG	GATGGGGGTCCCTGACCA	U53823.1
ZO-1	CGGGAAGTTACGTGGCGAAG	GTCAGCAGCACCCGTGG	NM_001330239.4
TGF β1	TTGACTTCCGCAAGGACCTC	GAAGTTGGCATGGTAGCCCT	NM_000660.7
IL-1β	AACCTCTTCGAGGCACAAGG	GCTGAAGAGAATCCCAGAGCA	NM_000576.3
IL-6	TCTGCGCAGCTTTAAGGAGT	GTGCCCATGCTACATTTGCC	NM_001371096.1
TNF α	TCCCCAGGGACCTCTCTCTA	CTTGTCACTCGGGGTTCGAG	NM_000594.4
GAPDH	CCACATCGCTCAGAACACCT	GCGCCCAATACGACCAAATC	M17851.1

## Data Availability

The original contributions presented in the study are included in the article, further inquiries can be directed to the corresponding author.

## Supplementary Materials

The following supporting information can be downloaded at: https://www.mdpi.com/article/10.3390/nu16234077/s1.
