# Supplementary figures and images for: Effects of Forming Lactoferrin–Milk Protein Complexes on Lactoferrin Functionality and Intestinal Development in Infancy
Source: Nutrients. 2024 Nov 27;16(23):4077. doi: 10.3390/nu16234077 (PMC11644007; doi:10.3390/nu16234077)

## Slide 1
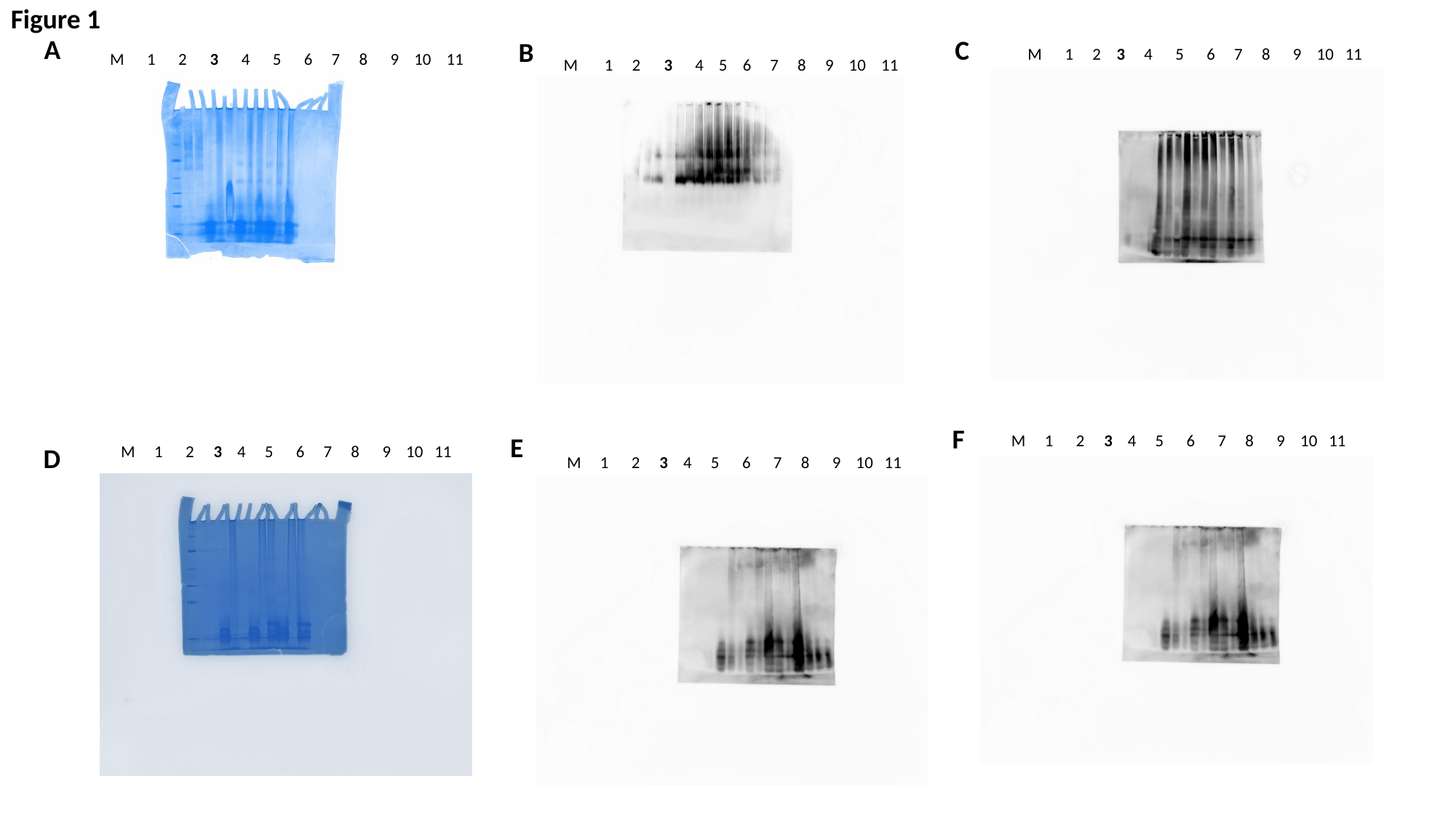

Figure 1
A
C
B
 M 1 2 3 4 5 6 7 8 9 10 11
 M 1 2 3 4 5 6 7 8 9 10 11
 M 1 2 3 4 5 6 7 8 9 10 11
F
E
 M 1 2 3 4 5 6 7 8 9 10 11
D
 M 1 2 3 4 5 6 7 8 9 10 11
 M 1 2 3 4 5 6 7 8 9 10 11

## Slide 2
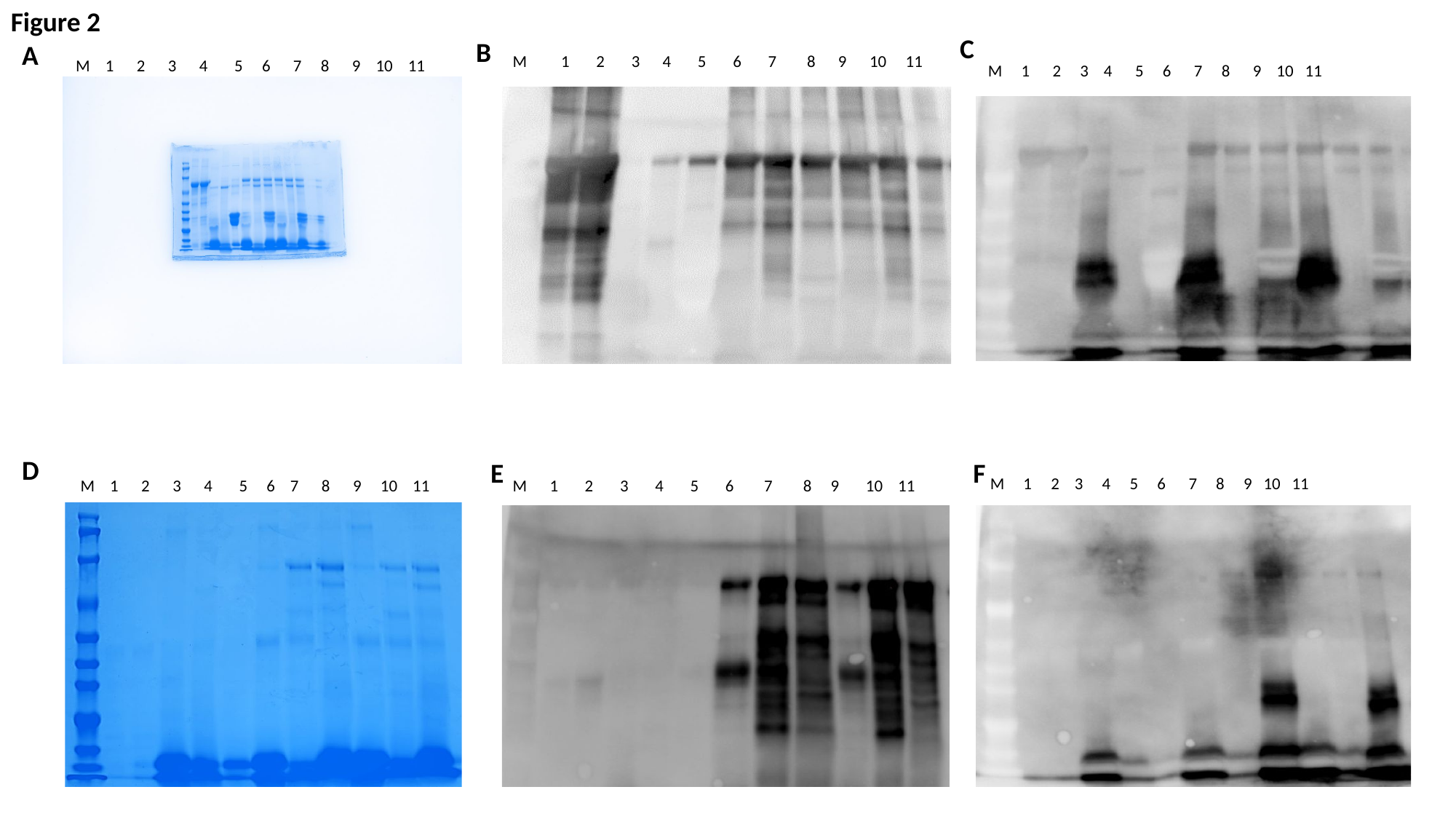

Figure 2
C
B
A
M 1 2 3 4 5 6 7 8 9 10 11
M 1 2 3 4 5 6 7 8 9 10 11
M 1 2 3 4 5 6 7 8 9 10 11
D
E
F
M 1 2 3 4 5 6 7 8 9 10 11
M 1 2 3 4 5 6 7 8 9 10 11
M 1 2 3 4 5 6 7 8 9 10 11

Supplement: Supplementary file 1 [file nutrients-16-04077-s001.zip › nutrients-3250005-supplementary.pptx]
